# Web-Based Parent Training With Telephone Coaching Aimed at Treating Child Disruptive Behaviors in a Clinical Setting During the COVID-19 Pandemic: Single-Group Study With 2-Year Follow-Up

**DOI:** 10.2196/63416

**Published:** 2024-12-16

**Authors:** Saana Sourander, Minja Westerlund, Amit Baumel, Susanna Hinkka-Yli-Salomäki, Terja Ristkari, Marjo Kurki, Andre Sourander

**Affiliations:** 1 Research Centre for Child Psychiatry, University of Turku, Turku, Finland; 2 INVEST Research Flagship Centre, University of Turku, Turku, Finland; 3 Department of Community Mental Health, University of Haifa, Haifa, Israel; ^4^ITLA Children's Foundation, Helsinki, Finland; 5 Department of Child Psychiatry, Turku University Hospital, Turku, Finland

**Keywords:** parent training, disruptive behavior, child psychopathology, child functioning, behaviors, behavioral, coaching, web-based, family counseling, child, disruptive, counseling, training, parents, parenting, telephone, telehealth, telemedicine, pediatrics, COVID-19

## Abstract

**Background:**

There is a lack of studies examining the long-term outcomes of web-based parent training programs implemented in clinical settings during the COVID-19 pandemic.

**Objective:**

The aim is to study 2-year outcomes of families with 3‐ to 8-year-old children referred from family counseling centers to the Finnish Strongest Families Smart Website (SFSW), which provides digital parent training with telephone coaching aimed at treating child disruptive behaviors.

**Methods:**

Counseling centers in Helsinki identified fifty 3‐ to 8-year-old children with high levels of disruptive behavioral problems. Child psychopathology and functioning as well as parenting styles and parental mental health were collected from parents at baseline; posttreatment; and at 6-, 12-, and 24-month follow-ups.

**Results:**

The SFSW program had positive long-term changes in child psychopathology and parenting skills. Improvements in child psychopathology, including Strengths and Difficulties Questionnaire total score (Cohen *d*=0.47; *P*<.001), Strengths and Difficulties Questionnaire conduct scores (Cohen *d*=0.65; *P*<.001), and Affective Reactivity Index irritability scores (Cohen *d*=0.52; *P*<.001), were maintained until the 24-month follow-up. Similarly, changes in parenting skills measured with the Parenting Scale, including overreactivity (Cohen *d*=0.41; *P*=.001) and laxness (Cohen *d*=0.26; *P*=.02), were maintained until the 24-month follow-up. However, parental hostility changes were not maintained at long-term follow-up (Cohen *d*=−0.04; *P*=.70).

**Conclusions:**

The study shows that the SFSW parent training program can yield significant long-term benefits. Findings indicate that the benefits of the treatment may vary between different parenting styles, which is important to consider when developing more personalized parenting interventions.

## Introduction

There is growing evidence from randomized controlled trials (RCTs) that parents can be trained to intervene and reduce child disruptive behaviors and improve their parenting skills [1-3]. Parent training has been found to be the most effective way to prevent and treat disruptive behaviors (patterns of, eg, defiant, disobedient, hostile, and externalizing behavior) among children [4-6] and is considered one of the most-validated therapeutic techniques in child mental health [7]. In the face of the unmet need for accessible evidence-based treatment programs to tackle early-onset childhood disruptive behaviors, digitally administered remote treatments provide solutions that require fewer personnel, that may be less stigmatizing, and that can reach geographically remote areas [8,9]. Examining the long-term outcomes of an intervention is an essential step in ensuring the sustainability of its effects. A recent meta-analysis on RCTs showed that parenting interventions based on social learning theory are effective in reducing physical and emotional violence at immediate posttest, but effects decrease over time [10]. However, to our knowledge, there are no long-term follow-ups (ie, more than 12 months) of digital parent training interventions in clinical settings. The Finnish Strongest Families Smart Website (SFSW) intervention is an 11-week program that includes parent training delivered through a digital platform and assisted by weekly telephone coaching. Previously, we conducted an RCT, screening families at regular health checkups for 4-year-old children. Children who had parent-reported high levels of disruptive behavior were screened for targeted SFSW intervention. The sample included 232 children in each study group, an intervention group, and an educational control group. At a 24-month follow-up after randomization, the results maintained efficacy in reducing a wide range of child psychopathology and improving parenting skills [11].

Child behavior problems are associated with psychological distress and financial costs and with a poor long-term outlook if they are not addressed [1]. Early intervention is crucial to help mitigate these impacts and support healthier development trajectories. This study targets an important gap in the current literature on long-term follow-ups of digital parent training interventions in clinical settings. We assessed the long-term outcomes of families participating in the SFSW parent training program in a clinical setting at baseline and at 12 and 24 months after baseline. A unique contribution of this study is that the SFSW parent training program was administered to the study cohort during the worst phase of the COVID-19 pandemic when the Helsinki capital region was partially isolated from other parts of Finland. Other services were severely limited, albeit the need for services was great. The SFSW program was an already existing, empirically tested, and, importantly, digitalized intervention targeting children’s disruptive behavior problems. Due to the crisis, it was considered ethically inappropriate to conduct a randomized controlled study design in this study. The program completion rate was high. The 6-month follow-up findings of this program were very promising and have been reported previously [12]. There were significant changes in most of the child psychopathology measures, including the Child Behavior Checklist (CBCL) externalizing score (mean 7.0, 95% CI 4.9‐9.0; *P*<.001), and when parenting skills were measured with the Parenting Scale (PS), the results showed significant changes in the total score (mean 0.5, 95% CI 0.4‐0.7; *P*<.001) [12].

Our aim with this paper is to report long-term changes in children’s functioning, psychopathology levels, parenting skills, and well-being from baseline until the 24-month follow-up. We hypothesized that the previously reported positive effects of the SFSW at the 6-month follow-up [12] would be at least partly maintained at the 24-month follow-up.

## Methods

### Study Population

The study included families with children between the ages of 3 and 8 years who exhibited elevated levels of disruptive behavior when screened by professionals from 8 different family counseling centers in Helsinki. Family counseling centers operate under social services and provide low-threshold services. The centers contribute to child development by reinforcing parenting skills and family relationships. They provide direct support, offer advice to assisting services, and facilitate referrals to specialized services. At family counseling centers, parent training that addresses child-rearing challenges is offered through individual or group sessions. The recruitment was based on identified needs. Health care and social welfare professionals from the counseling centers identified families that were in need of support for child disruptive behavior and selected families who were suitable for the remotely administered SFSW parent training program. During the pandemic lockdowns, face-to-face sessions were not possible, underscoring the importance of remote support methods.

### Ethical Considerations

Ethics approval for the study was received from the ethics committee of the University of Turku (statement 25/2018), and the study also received a research permit from the city of Helsinki (HEL 2020-006651). Parents provided written informed consent and were advised that participation in the study was voluntary and they had the right to withdraw at any time. This is a single-group study design with repeated measurements. Parents completed questionnaires at baseline; posttreatment; and at 6, 12, and 24 months after starting the program. The study encompassed 50 families and took place from May 2020 to November 2022. Earlier findings comparing baseline, posttreatment, and 6-month follow-up results have been reported previously [12].

### Recruitment, Eligibility, and Procedure

The professionals identified children with high levels of disruptive behavior problems. In addition, parents completed the Strengths and Difficulties Questionnaire (SDQ) [13,14] and were included if their child had a high level of conduct problems (≥5 points in the SDQ conduct scale) and if the parents perceived their child to have difficulties concerning emotions, behavior, or social interactions based on one item inquiring parents about these aspects. Additionally, inclusion in the study required at least 1 parent to be a native Finnish or Swedish speaker with access to both a telephone and a device with internet connectivity. Exclusion criteria were if a child had been diagnosed with autism, Down syndrome, fetal alcohol syndrome, an intellectual disability, or severe mental disorders, which would indicate a need for services beyond the scope of the digital SFSW program. Eligible families were invited to participate and directed to the SFSW website to provide their formal consent and fill in the baseline questionnaires. Commencing with the completion of baseline questionnaires, participants progressed through SFSW. A flowchart on the study procedures is provided in Multimedia Appendix 1.

### Intervention

The SFSW parent training program used digital materials (eg, psychoeducational material, video clips, and home exercises) and telephone coaching. The program focused on enhancing skills to improve parent-child relationships, complemented by weekly telephone sessions conducted by trained family coaches—licensed health care professionals, including nurses and public health nurses. The content of each web-based session included an introduction, session content, video exercises, troubleshooting, review, and practical application of the new skills. Each session featured multimedia components and digital exercises, and parents were encouraged to complete the session before the next phone call. All coaching calls were systematically recorded and subjected to random audits by the coach supervisor to ensure fidelity. The telephone sessions were scheduled at the end of the previous weekly call for a duration of 1 hour each. The family coach followed up in case the family missed an appointment, and possible rescheduling of coaching sessions was done per SMS text message or email contact by the parent.

The SFSW program has previously been shown to be effective [11,15] and can successfully make the transition to implementation settings [12,16,17]. Table 1 includes an outline of the weekly themes covered in SFSW. The program was sequential, that is, the parents completed 1-week theme before moving to the next. The primary goal was for parents to recognize positive behaviors in their children and respond positively. The second aim was to apply learned skills in everyday situations, using positive methods to reinforce the child’s positive behavior. The end of the program focused on solidifying the application of newfound positive parenting skills in daily life to support the child’s positive behavior. Parents practiced these skills with their children and discussed their progress during weekly telephone calls with their family coach, which were scheduled aiming to ensure sustainability beyond the program’s completion. As previously reported [12], the average time spent on the program website for each of the 11 themes was 48.0 (SD 25.6) minutes, and the mean duration of telephone coaching was 35.3 (SD 8.8) minutes per call. The total mean duration per theme, including both digital materials and telephone coaching, was 83.3 (SD 28.0) minutes.

**Table 1.** Themes of the SFSW^a^ web-based parent training program for children with behavioral problems.

Session	Goals
1. Notice the good	Boost the child’s self-esteem, boost the parent’s self-esteem, and change the parent’s view of their child
2. Spread attention around	Strengthen the child’s empathy skills
3. Ignore whining and complaining	Teach parents self-regulation
4. Prepare for changes	Reinforce good daily routines
5. Plan ahead at home	Boost the self-esteem of the child and parent and involve the child in planning
6. Chart and stickers	Involve the child in planning and reinforce good daily routines
7. Plan ahead outside the home	Boost the self-esteem of the child and parent and involve the child in planning
8. Working with daycare	Help the child to manage and succeed
9. Time-out	Teach self-regulation and consistency
10 and 11. Problem-solving revision and future application of skills	Teach parents skills to support child development and prepare for future challenges
12. Booster	Remind parents of positive proactive parenting skills

a SFSW: Strongest Families Smart Website.

### Measurements

The parents completed questionnaires at baseline, after the program, and at 6, 12, and 24 months after they had started the program. In addition, demographic details of the family, children, and parents were collected during the screening phase. All of the measurements used in this study have demonstrated adequate reliability and criterion validity metrics and were described more extensively in our previous paper [12]. For brevity, we mention them briefly below, while a comprehensive description is provided in Multimedia Appendix 2.

### Child Psychopathology and Functioning

Child psychopathology was assessed using the Finnish version of the 25-item SDQ [13,14], which measures challenges the child experiences in emotions, behavior, or social interactions [18]. Perceived difficulties were gauged through a single question regarding challenges in emotions, behavior, or social interactions, with response options ranging from no difficulties to severe difficulties. Disruptive behavior was gauged by the externalizing subscale of the CBCL for ages 1.5‐5 years (99 items) [19], focusing on an externalizing subscale with 24 items related to attention issues and aggressive behavior (our primary outcome) along with the CBCL’s total score. Child irritability was measured by the Affective Reactivity Index (ARI), which includes 6 irritability symptom items and 1 impairment item [20]. A 17-item questionnaire, derived from Barkley’s Home Situations Questionnaire [21], measured the parents’ experiences of their child’s functioning and behavior in daily situations. We used the 24-item Inventory of Callous-Unemotional Traits (ICU) [22] to assess 3 precursors of child psychopathy: callousness, uncaring, and unemotional traits [23,24].

### Parenting, Parental Mental Health, and Satisfaction

The PS, a 30-item tool, was used to evaluate 3 dysfunctional parenting discipline styles: laxness, overreactivity, and hostility, reflecting rule enforcement, responses to mistakes, and using verbal or physical force, respectively [25,26]. We used the 21-item Depression, Anxiety, and Stress Scale (DASS-21) to assess parental stress, anxiety, and depression symptoms in the past week [27].

### Statistical Analysis

Descriptive statistics include numbers and percentages for categorical variables and means and SDs for continuous variables. The categorical variables were analyzed with Pearson chi-square or Fisher exact tests and the continuous variables with 2-sample 2-tailed *t* test. We analyzed the outcome variables using linear mixed-effect models for repeated measurements with time as a within-factor. The modeling framework enables to use restricted maximum estimation method, which handles data with missing observations. Therefore, there was no need to apply any separate imputation method. We used linear contrasts to estimate changes from baseline to 12 and 24 months as well as changes from 12 to 24 months. We included the sex, age, and maternal education of the children as covariates in all models. The McNemar test was applied to test the change in the number of children with a total SDQ score above the 90th percentile (ie, abnormal range) at baseline and at the 24-month follow-up. The effect sizes of all outcome variables were calculated as 2-tailed *t* test effect sizes using Cohen *d*. The statistical analyses were performed using SAS statistical software (version 9.4; SAS Institute Inc).

## Results

### Participant Characteristics

The study comprised 50 families who were referred to the program, of which 44 (88%) completed the whole 11-week SFSW program. In total, 24-month follow-up assessments were obtained from 37 (74%) families. As shown in Table 2, 37 (74%) of the 50 children were boys. A total of 48 (96%) children had definitive or severe behavioral problems at baseline, and only 2 (4%) had minor behavioral problems based on a single item in the parent report, “Overall, do you think that your child has difficulties in 1 or more of the following areas: emotions, behavior, or being able to get on with other people?” Table 2 also presents a comparison between the families who completed the 24-month follow-up and those who did not. The table shows the difference in maternal education—in the noncompleter group, mothers were less educated. The completers and noncompleters did not differ on parenting style measures or psychopathology symptoms.

**Table 2.** Baseline characteristics of enrolled families, and comparison between families completing and those not completing the 24-month follow-up measurements.

Baseline characteristics			All families (n=50)	Families completing the 24-month follow-up (n=37)	Families not completing 24-month follow-up (n=13)	*P* value^a^
**Family and parent characteristics**						
	**Family structure, n (%)**					.21
		Biological parents	38 (76)	30 (81)	8 (62)	
		One biological parent	11 (22)	6 (16)	5 (38)	
		Other	1 (2)	1 (3)	0 (0)	
	**Age (years), mean (SD)**					
		Maternal	31.9 (4.3)	31.9 (3.5)	31.6 (6.2)	.86
		Paternal	32.8 (3.7)	32.3 (3.6)	34.5 (3.7)	.10
	**Maternal educational level^b^, n (%)**					.047
		College or university degree	38 (78)	31 (86)	7 (54)	
		Lower	11 (22)	5 (14)	6 (46)	
	**Paternal educational level^c^, n (%**)					.46
		College or university degree	32 (70)	23 (66)	9 (82)	
		Lower	14 (30)	12 (34)	2 (18)	
	**Mother’s native language, n (%)**					.55
		Finnish	45 (90)	34 (92)	11 (85)	
		Swedish	3 (6)	2 (5)	1 (8)	
		Other	2 (4)	1 (3)	1 (8)	
	**Father’s native language^c^, n (%)**					.21
		Finnish	37 (80)	29 (83)	8 (73)	
		Swedish	3 (7)	1 (3)	2 (18)	
		Other	6 (13)	5 (14)	1 (9)	
	**Parenting Scale, mean (SD)**					
		Total	3.5 (0.5)	3.5 (0.5)	3.5 (0.3)	.81
		Laxness	2.8 (0.8)	2.8 (0.8)	2.8 (0.7)	.95
		Overreactivity	4.3 (1.2)	4.1 (1.2)	4.6 (1.2)	.25
		Hostility	1.9 (0.8)	1.8 (0.8)	1.9 (0.8)	.71
	**DASS-21^d^, mean (SD)**					
		Total	22.6 (14.9)	20.8 (14.2)	28.0 (16.3)	.13
		Depression	6.6 (7.1)	5.3 (5.8)	10.5 (9.1)	.07
		Anxiety	2.8 (4.3)	2.9 (4.3)	2.6 (4.6)	.86
		Stress	13.2 (6.6)	12.6 (6.9)	14.9 (5.6)	.28
**Child characteristics**						
	**Sex, n (%)**					.14
		Female	13 (26)	12 (32)	1 (8)	
		Male	37 (74)	25 (68)	12 (92)	
	**Age (years), n (%)**					.33
		3‐5	30 (60)	24 (65)	6 (46)	
		6‐8	20 (40)	13 (35)	7 (54)	
	**Behavioral problems, n (%)**					.43
		Minor	2 (4)	2 (5)	0 (0)	
		Definite	30 (60)	20 (54)	10 (77)	
		Severe	18 (36)	15 (41)	3 (23)	
	**CBCL/1.5‐5^e^, mean (SD)**					
		Total	62.1 (22)	63.6 (21.9)	57.7 (22.6)	.41
		Externalizing	25.7 (7)	26.0 (6.9)	24.9 (7.6)	.63
	**SDQ^f^, mean (SD)**					
		Total	19.8 (4.8)	20.2 (4.6)	18.5 (5.1)	.26
		Emotional	3.5 (2.3)	3.7 (2.2)	3.0 (2.5)	.34
		Conduct	7.5 (1.4)	7.5 (1.4)	7.2 (1.5)	.50
		Hyperactivity	6.0 (2.4)	6.1 (2.3)	5.5 (2.5)	.46
		Peer	2.8 (1.9)	2.9 (1.9)	2.7 (2.0)	.78
		Prosocial	5.2 (2.0)	5.1 (2.1)	5.2 (1.7)	.88
		Impact	3.0 (1.7)	3.0 (1.6)	2.9 (2.1)	.93
	**ARI^g^, mean (SD)**					
		Irritability	8.6 (3.2)	8.7 (3.0)	8.4 (3.7)	.74
	**ICU^h^, mean (SD)**					
		Total	27.4 (7.7)	26.5 (7.3)	30.1 (8.6)	.16
		Callousness	8.9 (3.6)	8.5 (3.5)	10.0 (3.8)	.19
		Uncaring	14.5 (3.5)	14.2 (3.6)	15.2 (3.3)	.37
		Unemotional	4.1 (3.0)	3.8 (2.7)	4.8 (3.8)	.30
	**Everyday situations (child behavior), mean (SD)**					
		Total	42.9 (11.3)	43.8 (11.2)	40.3 (11.7)	.34
		Transition situations	14.7 (4.4)	14.9 (4.4)	14.2 (4.7)	.65
		Dining situations	7.8 (3.0)	8.1 (3.0)	7.0 (2.9)	.27
		Situations outside home	10.4 (3.3)	10.8 (3.3)	9.5 (3.3)	.23
		Home situations	10.0 (3.1)	10.1 (3.1)	9.6 (3.5)	.63

a Refers to statistical test comparing families completing the 24-month follow-up to those who did not.

b One missing observation.

c Four missing observations.

d DASS-21: 21-item Depression, Anxiety, and Stress Scale.

e CBCL/1.5‐5: Child Behavior Checklist for preschool children.

f SDQ: Strengths and Difficulties Questionnaire.

g ARI: Affective Reactivity Index.

h ICU: Inventory of Callous-Unemotional Traits.

### Long-Term Changes in Child and Parenting Measures

Descriptive statistics of child psychopathology, child function level, parental skills, and parental mental health at baseline and at 12 and 24 months after baseline are presented in Table 3. A statistical comparison of the different time points is presented in Table 4. In terms of child psychopathology, significant improvements between baseline and the 12-month follow-up as well as between baseline and the 24-month follow-up were found in CBCL total scores and externalizing scores, SDQ total scores and most subscales (emotional, conduct, hyperactivity, and peer problems), and irritability measured with the ARI scale. At the same time, there was a significant deterioration in CBCL total and externalizing scores and SDQ prosocial behavior scores between the 12-month and 24-month follow-ups.

**Table 3.** Child psychopathology, child functioning level, parental skills, and parental mental health at baseline and 12 months and 24 months after the baseline (n=50).

Variable			Baseline^a^, mean^b^ (SE)	12 months^c^, mean (SE)	24 months^d^, mean (SE)
**Child psychopathology**					
	**CBCL/1.5-5** ^**e**^				
		Total	61.8 (5.5)	43.8 (5.8)	50.2 (5.9)
		Externalizing	25.5 (1.9)	18.0 (2.2)	20.2 (2.2)
	**SDQ** ^**f**^				
		Total	19.8 (1.1)	14.4 (1.3)	15.3 (1.3)
		Emotional symptoms	3.5 (0.5)	2.1 (0.5)	2.7 (0.5)
		Conduct problems	7.3 (0.4)	5.1 (0.4)	5.2 (0.5)
		Hyperactivity	6.8 (0.6)	5.5 (0.7)	5.7 (0.7)
		Peer problems	2.1 (0.4)	1.5 (0.4)	1.5 (0.5)
		Prosocial behavior	5.6 (0.5)	6.4 (0.5)	5.8 (0.5)
		Impact	3.2 (0.4)	2.0 (0.4)	2.7 (0.4)
	**ARI** ^**g**^				
		Irritability	9.3 (0.8)	6.2 (0.8)	6.4 (0.8)
	**ICU** ^**h**^				
		Total	25.9 (1.8)	22.5 (2.0)	24.1 (2.0)
		Callousness	8.1 (0.8)	6.2 (0.9)	6.1 (1.0)
		Uncaring	14.0 (0.8)	12.0 (0.9)	13.3 (0.9)
		Unemotional	4.2 (0.7)	4.6 (0.7)	5.1 (0.8)
**Child functioning level**					
	**Everyday situations**				
		Child behavior—total	42.4 (2.7)	33.4 (3.0)	33.6 (3.0)
		Transition situations	13.9 (1.1)	10.5 (1.1)	11.1 (1.1)
		Dining situations	7.8 (0.7)	6.7 (0.7)	6.4 (0.7)
		Situations outside home	10.3 (0.8)	8.0 (0.8)	7.8 (0.8)
		Home situations	10.3 (0.8)	8.0 (0.9)	8.2 (0.9)
**Parental skills**					
	**Parenting Scale**				
		Total	3.5 (0.1)	3.1 (0.1)	3.2 (0.1)
		Laxness	2.8 (0.2)	2.4 (0.2)	2.5 (0.2)
		Overreactivity	4.4 (0.3)	3.6 (0.3)	3.8 (0.3)
		Hostility	2.2 (0.2)	1.9 (0.2)	2.2 (0.2)
**Parental mental health**					
	**DASS-21** ^**i**^				
		Total	24.4 (3.8)	20.3 (3.9)	23.5 (4.0)
		Depression	8.2 (1.5)	6.7 (1.5)	7.7 (1.5)
		Anxiety	2.7 (1.0)	2.9 (1.1)	3.4 (1.2)
		Stress	13.5 (1.7)	10.6 (1.8)	12.5 (1.9)

a Measurements before the program started.

b Least-squares means.

c Measurements at 12 months after starting the program.

d Measurements at 24 months after starting the program.

e CBCL/1.5‐5: Child Behavior Checklist for preschool children.

f SDQ: Strengths and Difficulties Questionnaire.

g ARI: Affective Reactivity Index.

h ICU: Inventory of Callous-Unemotional Traits.

i DASS-21: 21-item Depression, Anxiety, and Stress Scale.

**Table 4.** Changes from baseline to 12 months and 24 months after in child psychopathology, child function level, parental skills, and parental mental health.

Variable			Baseline^a^ to 12 months^b^			Baseline to 24 months^c^			12 months to 24 months	
			Mean (95% CI)	*P* value	Cohen *d*	Mean (95% CI)	*P* value	Cohen *d*	Mean (95% CI)	*P* value
**Child psychopathology**										
	**CBCL/1.5-5** ^**d**^									
		Total	17.4 (9.8 to 25.0)	<.001	0.49	11.0 (3.5 to 18.5)	.005	0.32	−6.4 (10.2 to −2.7)	.001
		Externalizing	7.5 (4.7 to 9.9)	<.001	0.66	5.2 (2.4 to 8.1)	.001	0.39	−2.3 (−4.1 to −0.4)	.02
	**SDQ** ^**e**^									
		Total	5.4 (3.5 to 7.3)	<.001	0.62	4.5 (2.4 to 6.5)	<.001	0.47	−0.9 (−2.1 to 0.3)	.34
		Emotional	1.4 (0.7 to 2.1)	<.001	0.43	0.8 (−0.0 to 1.7)	.053	0.21	−0.6 (−1.1 to −0.1)	.03
		Conduct	2.2 (1.6 to 2.8)	<.001	0.78	2.1 (1.4 to 2.8)	<.001	0.65	−0.1 (−0.7 to 0.5)	.68
		Hyperactivity	1.2 (0.5 to 2.0)	.002	0.34	1.0 (0.3 to 1.8)	.008	0.29	−0.2 (−0.8 to 0.3)	.44
		Peer	0.6 (0.1 to 1.0)	.02	0.25	0.6 (0.1 to 1.2)	.03	0.24	0.1 (−0.4 to 0.6)	.77
		Prosocial^f^	−0.8 (−1.4 to −0.2)	.009	−0.27	−0.2 (−0.8 to 0.4)	.45	−0.08	0.6 (0.1 to 1.1)	.02
		Impact	1.2 (0.6 to 1.8)	<.001	0.45	0.5 (−0.2 to 1.1)	.15	0.16	−0.7 (−1.3 to −0.2)	.01
	**ARI** ^**g**^									
		Irritability	3.1 (1.9 to 4.3)	<.001	0.57	2.9 (1.7 to 4.1)	<.001	0.52	−0.2 (−1.4 to 1.0)	.71
	**ICU** ^**h**^									
		Total	3.4 (0.9 to 5.9)	.008	0.30	1.7 (−0.8 to 4.2)	.17	0.15	−1.7 (−3.7 to 0.4)	.11
		Callousness	1.9 (0.6 to 3.2)	.004	0.31	1.9 (0.5 to 3.4)	.008	0.29	0.0 (−1.2 to 1.3)	.94
		Uncaring	2.0 (0.7 to 3.2)	.003	0.33	0.8 (−0.3 to 1.8)	.17	0.15	−1.2 (−2.3 to −0.2)	.03
		Unemotional	−0.4 (−1.0 to 0.1)	.14	−0.16	−0.9 (−1.8 to 0.0)	.04	−0.22	−0.5 (−1.2 to 0.2)	.14
**Child’s ability to function**										
	**Everyday situations (child behavior)**									
		Child behavior total	9.1 (5.3 to 12.9)	<.001	0.52	8.9 (4.9 to 12.8)	<.001	0.49	−0.2 (−3.3 to 3.0)	.91
		Transition situations	3.4 (2.0 to 4.9)	<.001	0.52	2.8 (1.2 to 4.4)	.001	0.38	−0.6 (−1.7 to 0.5)	.27
		Dining situations	1.0 (0.2 to 1.9)	.01	0.28	1.4 (0.6 to 2.3)	.001	0.37	0.4 (−0.4 to 1.2)	.36
		Situations outside home	2.3 (1.1 to 3.4)	<.001	0.43	2.6 (1.4 to 3.7)	<.001	0.49	0.3 (−0.7 to 1.2)	.54
		Home situations	2.2 (1.1 to 3.3)	<.001	0.45	2.0 (0.9 to 3.2)	.001	0.40	−0.2 (−1.2 to 0.9)	.73
**Parental skills**										
	**Parenting Scale**									
		Total	0.5 (0.3 to 0.6)	<.001	0.68	0.4 (0.2 to 0.5)	<.001	0.49	−0.1 (−0.2 to 0.0)	.15
		Laxness	0.4 (0.1 to 0.6)	.002	0.35	0.3 (0.1 to 0.5)	.02	0.26	−0.1 (−0.4 to 0.1)	.39
		Overreactivity	0.8 (0.5 to 1.1)	<.001	0.59	0.6 (0.3 to 0.9)	.001	0.41	−0.2 (−0.4 to 0.1)	.09
		Hostility	0.2 (0.0 to 0.3)	.01	0.28	−0.0 (−0.3 to 0.2)	.70	−0.04	−0.2 (−0.5 to −0.0)	.03
**Parental mental health**										
	**DASS-21** ^**i**^									
		Total	4.0 (−0.8 to 8.9)	.10	0.18	0.9 (−4.2 to 5.9)	.73	0.04	−3.2 (−8.3 to 1.9)	.22
		Depression	1.5 (−0.7 to 3.8)	.17	0.15	0.6 (−1.4 to 2.6)	.57	0.06	−1.0 (−2.8 to 0.8)	.29
		Anxiety	−0.2 (−1.7 to 1.2)	.74	0.04	−0.7 (−2.7 to 1.2)	.45	0.08	−0.5 (−2.0 to 1.0)	.51
		Stress	2.9 (0.7 to 5.0)	.009	0.29	1.0 (−1.1 to 3.2)	.34	0.10	−1.9 (−4.3 to 0.6)	.14

a Measurement before the program started.

b Measurement at 12 months after the program started.

c Measurement at 24 months after the program started.

d CBCL/1.5‐5: Child Behavior Checklist for preschool children.

e SDQ: Strengths and Difficulties Questionnaire.

f Increase in prosocial SDQ subscore indicates improvement.

g ARI: Affective Reactivity Index.

h ICU: Inventory of Callous-Unemotional Traits.

i DASS-21: 21-item Depression, Anxiety, and Stress Scale.

We conducted an additional analysis of 37 (74%) of the 50 parents who completed the SDQ questionnaire both at baseline and at the 24-month follow-up as well as the parent training program. This analysis showed that 30 (81%) of the 37 children had a total SDQ score above the 90th percentile (ie, abnormal range) at baseline, while only 14 (38%) remained in the abnormal range at the 24-month follow-up (*P*<.001, McNemar test), based on the population sample of 4‐ to 16-year-old children [12]. To examine the children in the proximity of cutoff thresholds, we also used the 80th percentile cutoff point (ie, abnormal or border range), which showed that 36 (97%) children were above the cutoff point at baseline, while the respective figure at the 24-month follow-up was 23 (62%), indicating a highly significant change (*P*<.001, McNemar test).

When parents were asked about perceived difficulties regarding their child’s behavior problems with a single question—“Overall, do you think that your child has difficulties in 1 or more of the following areas: emotions, behavior, or being able to get on with other people?”—at baseline, 2 (5%) of 37 had no or minor problems, 20 (54%) had definite problems, and 15 (41%) had severe problems. The respective figures at the 24-month follow-up were 14 (38%), 14 (38%), and 9 (24%; *P*=.001, McNemar-Bowker test).

Among the child psychometric measures, callousness and uncaring improved between baseline and the 12-month follow-up. However, uncaring deteriorated between the 12- and 24-month follow-up, and no significant improvement was found between baseline and 24 months. The SFSW parent training program did not have any significant positive association with unemotional traits at the 12- or 24-month follow-up.

Child functioning in everyday situations (eg, transitions, dining, and home and outside home activities) improved significantly between baseline and both follow-up points. No significant change was observed between the 12- and 24-month follow-up comparisons.

Interestingly, there were differences between parenting styles regarding the long-term changes. Parental overreactivity and laxness improved between baseline and the 12-month follow-up and between baseline and the 24-month follow-up. Parental hostility showed improvement between baseline and the 12-month follow-up but not between baseline and the 24-month comparison. In fact, hostility showed significant deterioration between the 12- and 24-month follow-up. We observed no significant association in parental mental health problems, measured with a 21-item Depression, Anxiety, and Stress Scale, between baseline and either of the follow-ups.

**Figure 1.**
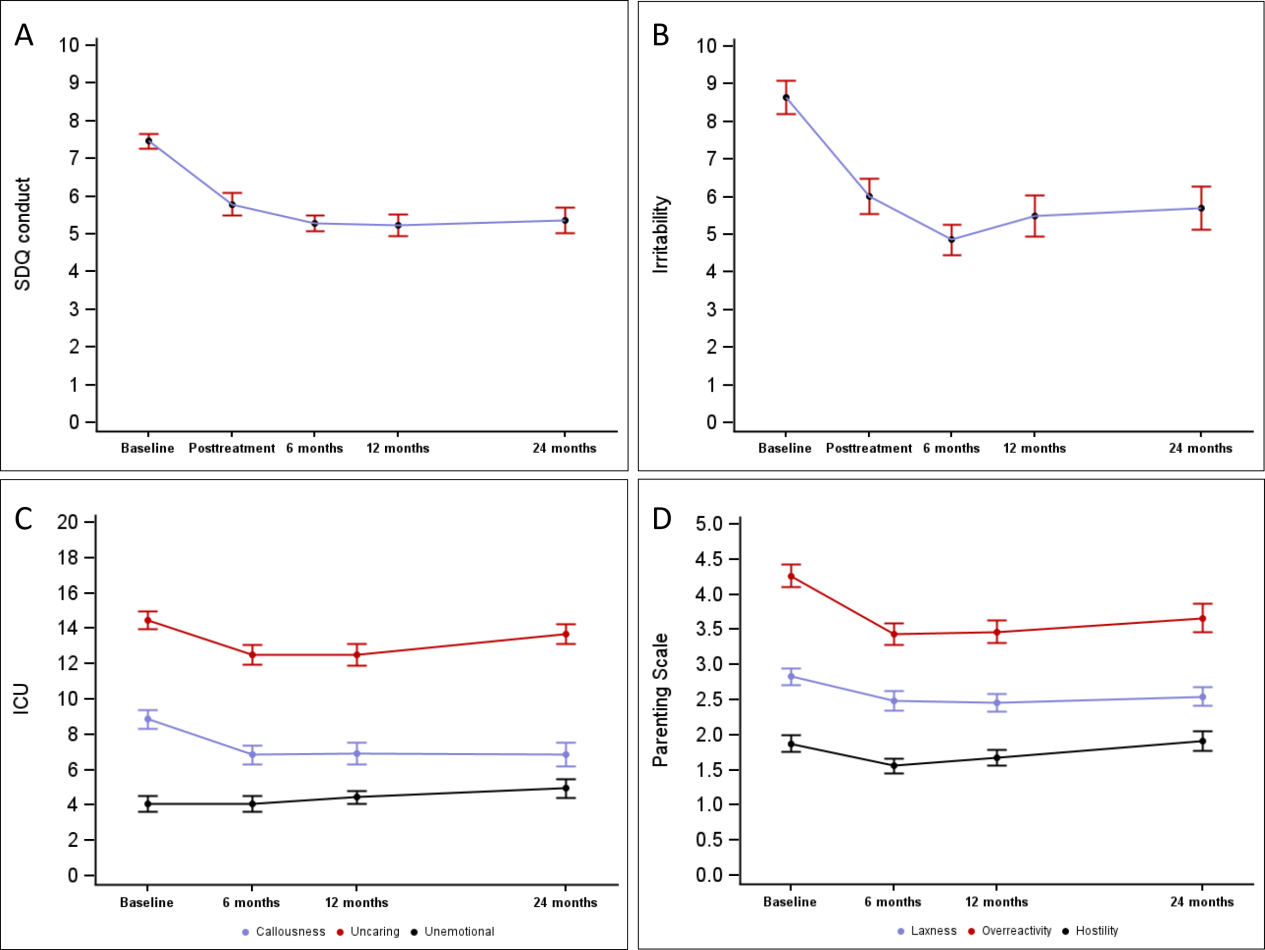
(A-D) Mean curves of SDQ conduct scores, irritability scores, ICU scores, and Parenting Scale subscores. (A) SDQ conduct scores over time (model-based least-squares means, SE). (B) Irritability scores over time (model-based least-squares means, SE). (C) ICU subscales over time (model-based least-squares means, SE). (D) Parenting Scale subscores over time (model-based least-squares means, SE). ICU: Inventory of Callous-Unemotional Traits; SDQ: Strengths and Difficulties Questionnaire.

Finally, to graphically illustrate the key findings, Figure 1A-D describes the changes of main outcome measures across time points including posttreatment and at the 6-month follow-up, which has previously been reported in detail [12]. Of note, the PS and ICU were not measured at posttreatment. The figures illustrate that SDQ conduct and ARI irritability scores exhibited the largest improvement between baseline and posttreatment and further improvement between posttreatment and the 6-month follow-up; the findings at the 12- and 24-month follow-ups were rather stable. Among the ICU measures, callousness and uncaring showed improvement between baseline and the 6-month follow-up. After that, callousness stayed quite stable, while uncaring and unemotional showed deterioration. As for the parenting measures, all parenting styles showed improvement between baseline and the 6-month follow-up. After that, overreactivity and laxness were quite stable, while hostility showed deterioration.

## Discussion

### Principal Findings

To the best of our knowledge, this is the first study on long-term follow-up of digital-guided parent training intervention among children referred to treatment from specialized care. The findings mostly complement the previously conducted 6-month follow-up study [12] by showing that the SFSW program was associated with significant improvements in children’s externalizing symptoms (our primary outcome) at 12 and 24 months after baseline. Of note, most of the improvement took place between baseline and posttreatment assessment, and the level of externalizing problems showed stability from the 12- to the 24-month follow-up. This study’s importance is in demonstrating that digital parent training with weekly remote phone coaching seems to lead to enduring improvements in disruptive behavior problems in children with severe disruptive behavior problems. The findings align with a 24-month follow-up study of the SFSW program [11,15-17], which was used as a preventive and early intervention among 4-year-old children, identified through national medical checkups [28]. However, since the target group was different, the level of disruptive behavior problems among the children in this study was much more severe [12], which suggests the promise of such interventions in supporting populations with different levels of symptom severity.

Most comorbidities such as hyperactivity, emotional and peer problems, and child functioning in everyday situations maintained their improvement from baseline to the 12- and 24-month follow-ups as well. Interestingly, the program seemed to have a very clear association with decreased irritability. This novel finding implies that some of the major effects of parent training may be associated with decreasing irritability in parent-child interactions; this requires further research.

For some problems, such as callous-unemotional traits, improvement was reported at the 12-month follow-up but not at the 24-month follow-up. Callous-unemotional traits characterize a specific subgroup of children exhibiting early starting, stable, and severe conduct problems. It has been argued that conventional parenting interventions frequently prove ineffective within this subgroup [29,30].

Another important finding was that the responses to the parent training program seemed to differ according to parenting styles. Improvements in parent overreactivity and laxness were shown both in the comparison between baseline and the 12- and 24-month follow-ups, while parental hostility improved until the 12-month follow-up, then deteriorated to the same level as at baseline. It could be interpreted that parental hostility is, in the long run, resistant to parent training programs. It is also possible that accumulated stressors during the COVID-19–related public health restrictions may have posed an additional strain on some parents’ psychological resources [31-33] and on their ability to maintain positive approaches to their child.

There is limited research on the effects of parenting interventions on reducing parental hostility. Parental hostility can have broad impacts within the family, potentially disrupting the ability of one parent to maintain a positive relationship with their child [34]. There is a significant positive correlation between parent hostility and child aggression, indicating that the more hostile parents are toward others, the more aggressive their children tend to be [35]. Similar findings regarding conduct problems, callous-unemotional traits, and parenting were made in a previous study [36], where higher levels of parental harshness were related to higher levels of child conduct problems and callous-unemotional traits. Children subjected to abuse resulting from their parents’ aggressive behavior may experience adverse effects on their self-control and exhibit challenges in impulse control by acting impulsively, speaking before thinking, and demonstrating a reduced capacity to tolerate frustration or cope with failure [37]. There are also findings showing that parental attitudes play a substantial role in the gradual enhancement of a child’s self-control, exerting a significant impact on the individual [35]. In cases where parent training proves to be ineffective in the long term in reducing parental hostility, the parent could benefit from receiving personal support or therapy to address this issue. It is likely that more tailor-made and targeted interventions and treatment plans would benefit this subgroup of families, which are at risk of falling into this kind of negative cycle.

When the study started in May 2020, Helsinki was grappling with the peak of the COVID-19 pandemic, marked by a state of emergency declared nationwide in Finland. Stringent social distancing measures were enforced in the region to curb the virus’s transmission, significantly affecting families residing in the area. The COVID-19 pandemic has highlighted the importance of exploring remote, digital, or digitally assisted solutions for ensuring that young children, and their families, are provided with prompt support for mental health problems. This study demonstrated that technology can provide effective alternatives to traditional face-to-face interventions and can overcome a number of barriers during crises. Technology can be used to provide the right treatment at the right time, with high levels of support and fidelity, providing greater access and convenience and requiring fewer costs and less time.

### Limitations

It is important to acknowledge certain limitations. The present results from our clinical sample do not necessarily generalize across community samples. Since no a priori power analysis was performed, we cannot exclude the possibility that the study was underpowered. We note that with a sample size of 50 participants, we had 80% power to detect an effect size of 0.35 (1-tailed, α=.05). The study design did not allow for direct conclusions regarding the program’s efficacy, as it lacked an intervention-control group design. However, the COVID-19 pandemic meant that treatment and family counseling services could not be provided in the usual way, and conducting a randomized controlled study design would have been considered ethically inappropriate. Due to the lack of a control group, it is not possible to exclude the possibility of some age-related spontaneous improvement reflected in the results. Nevertheless, an earlier RCT using population-based screening in Finland showed that the SFSW intervention was effective at 2-year follow-up [11]. Furthermore, the constraints of social distancing, which included school closures, also prevented us from conducting direct observations of parenting, clinical assessments, and teacher ratings. It is possible that some consequences of those restraints are also reflected in some of our measures, such as the SDQ prosocial and peer subscales.

### Conclusions

This study provides support for the utility of remotely delivered parent training interventions. Incorporating remote interventions into child mental health services also serves as a safeguard during crisis situations such as COVID-19.

The study shows that remote digital child mental health services bring substantial benefits to families that can last for up to 2 years. Overall, the study emphasizes that guided digital parent training programs can be a crucial component in developing evidence-based treatment practices for children and families.

The study also emphasizes the importance of conducting long-term follow-ups to understand long-term intervention gains. The study results indicate that different parenting profiles and child psychopathology may have varying effects on the long-term outcome of the program. This finding is important when developing personalized parenting interventions for increased impact.

## Supplementary material

10.2196/63416 Multimedia Appendix 1 Flowchart of study procedures.

10.2196/63416 Multimedia Appendix 2 Comprehensive description of measurements.

## Abbreviations

ARI: Affective Reactivity Index
CBCL: Child Behavior Checklist
ICU: Inventory of Callous-Unemotional Traits
PS: Parenting Scale
RCT: randomized controlled trial
SDQ: Strengths and Difficulties Questionnaire
SFSW: Strongest Families Smart Website
