# Supplementary material for: Web-Based Parent Training With Telephone Coaching Aimed at Treating Child Disruptive Behaviors in a Clinical Setting During the COVID-19 Pandemic: Single-Group Study With 2-Year Follow-Up
Source: JMIR Pediatr Parent. 2024 Dec 16;7:e63416. doi: 10.2196/63416 (PMC11683509; doi:10.2196/63416)
Supplement: Multimedia Appendix 1 [file pediatrics-v7-e63416-s001.docx]

The professionals working at the family counseling centers identify the families

Parents fill in the SDQ-questionnaire

The family coach places a recruitment call to the family.

Parents give informed consent and fill in the baseline questionnaires on the platform.

SFSW parent training program begins

11 Sessions

Weekly telephone calls

At the end of the program the parents fill in the posttreatment questionnaires

Feedback of the program provided the to families and professionals

6-month follow-up

Booster call by family coaches

12-month follow-up assessment

24-month follow-up assessment
