# Supplementary material for: Web-Based Parent Training With Telephone Coaching Aimed at Treating Child Disruptive Behaviors in a Clinical Setting During the COVID-19 Pandemic: Single-Group Study With 2-Year Follow-Up
Source: JMIR Pediatr Parent. 2024 Dec 16;7:e63416. doi: 10.2196/63416 (PMC11683509; doi:10.2196/63416)
Supplement: Multimedia Appendix 2 [file pediatrics-v7-e63416-s002.docx]

The parents completed online questionnaires at baseline, after the program and 6, 12 and 24 months after they had started the program. A comprehensive description about the measurements used in this study are described more extensively here.

***Demographic and family information***

Demographic details of the family and parents were collected during the screening phase, encompassing the child's gender, family structure, and the parents' birth year, native language, educational attainment, and employment status. The outcomes of the demographic and family information are presented in the Results section.

***Child psychopathology and functioning***

Psychopathology was assessed using the Finnish version of the SDQ [1,2], a brief behavioral screening questionnaire examining positive and negative behaviors in 3-16-year-olds. The SDQ includes 25 items across 5 subscales, rated on scale with 3-point scale. Perceived difficulties were gauged through a single question regarding challenges in emotions, behavior, or social interactions, with response options ranging from no difficulties to severe difficulties. One study reported an SDQ internal consistency score of 0.58 when used by preschool children's parents [3].

Disruptive behavior was gauged by the externalizing subscale of the Child Behavior Checklist-Parent Report Form (CBCL) for ages 1.5-5 years with 99 items grouped into subscales [4]. Focusing on the externalizing subscale with 24 items related to attention issues and aggressive behavior along with the CBCL's total score, parents assessed their child's behavior over the past 2 months using a 3-point scale. CBCL demonstrated good test-retest reliability (e.g., 0.81) and criterion validity (e.g., 0.56-0.87) [4].

Child irritability was measured by the Affective Reactivity Index (ARI) scale, which includes 6 irritability symptom items and 1 impairment item [5]. Parents evaluated their child's behavior over the past 6 months compared to same-age peers, responding to statements about irritability with not true (0 points), somewhat true (1 point), or certainly true (2 points). The ARI scale also inquired about the impact of irritability on the child.

A 17-item questionnaire, derived from Barkleys' Home Situation Questionnaire [6], measured parents' experiences of their child's functioning and behavior in daily situations. Questions covered behavior at home, during transitions, and while eating, with responses on a 5-point scale from easy (1 point) to awkward (5 points).

The 24-item Inventory of Callous-Unemotional Traits (ICU) [7] assessed three precursors of psychopathy: callousness, uncaring, and unemotional traits, using a 4-point Likert scale. Higher scores indicated elevated callous and emotional traits. ICU has been proven to be an important measure for identifying subgroups of aggressive and antisocial children and adolescents [8,9].

***Parenting, parental mental health and satisfaction***

The Parenting Scale (PS), a 30-item tool, evaluates parenting and discipline styles for children aged 1-12 years, emphasizing to those related to disruptive behavior [10,11]. The scale examines three dysfunctional discipline styles: laxness (11 items), overreactivity (10 items), and hostility (7 items), reflecting rule enforcement, responses to mistakes, and using verbal or physical force, respectively. Rated on a 7-point scale from ineffective to effective, the PS is commonly used to evaluate parent training programs. Parents reflected on their parenting skills over the preceding 2 months.

Parental stress, anxiety, and depression symptoms in the past week were assessed using the 21-item Depression, Anxiety, and Stress Scale (DASS-21) [12]. The three DASS-21 scales, each with 7 items, measure dysphoria, hopelessness, lack of interest (depression scale), situational anxiety, autonomic arousal, skeletal muscle effects (anxiety scale), and chronic nonspecific arousal (stress scale). Responses on a 4-point Likert scale ranged from 0 (did not apply to me at all) to 3 (applied to me very much or most of the time).

**References**

1. Goodman R (1997) The Strengths and Difficulties Questionnaire: a research note. J Child Psychol Psychiatry 38(5):581-586. https://doi.org/10.1111/j.1469-7610.1997.tb01545.x
2. Koskelainen M, Sourander A, Kaljonen A (2000) The Strengths and Difficulties Questionnaire among Finnish school-aged children and adolescents. Eur Child Adolesc Psychiatry 9(4):277-284. <https://doi.org/10.1007/s007870070031>
3. Klein AM, Otto Y, Fuchs S, Zenger M, Von Klitzing K (2013) Psychometric properties of the parent-rated SDQ in preschoolers. Eur J Psychol Asses 29(2):96-104. https://doi.org/10.1027/1015-5759/a000129
4. Achenbach TM, Rescorla LA (2000) Manual for the ASEBA Preschool Forms & Profiles. Burlington, VT.
5. Stringaris A, Goodman R, Ferdinando S, Razadan V, Muhere E, Leibenluft E, Britman MA (2012) The Affective Reactivity Index: a concise irritability scale for clinical and research settings. J Child Psychol Psychiatry 53(11):1109-1117. https://doi.org/10.1111/j.1469-7610.2012.02561.x
6. Barkley RA, Murphy KR (1998) Home situations questionnaire. In: Attention-Deficit Hyperactivity Disorder: A Clinical Workbook, 2nd Edition. New York City, New York.
7. Frick PJ (2004) Inventory of Callous-Unemotional Traits. Department of Psychology, University of New Orleans. https://faculty.lsu.edu/pfricklab/icu.php. Accessed 26 March 2024.
8. Essau CA, Sasagawa S, Frick PJ (2006) Callous-unemotional traits in a community sample of adolescents. Assessment 13(4):454-469. https://doi.org/10.1177/1073191106287354
9. Fanti KA, Frick PJ, Georgiou S (2008) Linking callous-unemotional traits to instrumental and non-instrumental forms of aggression. J Psychopathol Behav Assess 31(4):285-298. https://doi.org/10.1007/s10862-008-9111-3
10. Arnold DS, O´Leary SG, Wolff LS, Acker MM (1993) The parenting Scale: A measure of dysfunctional parenting in discipline situations. Psychol Asses 5(2):137-144. https://doi.org/10.1037/1040-3590.5.2.137
11. Rhoades KA, O´Leary SG (2007) Factor structure and validity of parenting scale. J Clin Child Adolesc Psychol 36(2):137-146. https://doi.org/10.1080/15374410701274157
12. Lovibond SH, Lovibond PF (1995) Manual for the Depression Anxiety Stress Scales. 2nd ed. Sydney, NSW.
